# LTM-UNet: Linear Transformer–Mamba with Attention-Based U-Net for Context-Aware Breast Ultrasound Image Segmentation

**DOI:** 10.3390/diagnostics16121888

**Published:** 2026-06-17

**Authors:** Shivpratap Singh Kushwah, Santosh Prakash Chouhan, Narinder Singh Punn, Mahua Bhattacharya

**Affiliations:** 1 Department of Information Technology, Atal Bihari Vajpayee Indian Institute of Information Technology and Management, Gwalior 474015, MP, India; shivpratap@iiitm.ac.in (S.S.K.); santoshc@iiitm.ac.in (S.P.C.); mb@iiitm.ac.in (M.B.); 2 Centre for Biomedical Research, Atal Bihari Vajpayee Indian Institute of Information Technology and Management, Gwalior 474015, MP, India

**Keywords:** breast lesion, transformer, attention-guided skip fusion, state-space model, ultrasound medical imaging

## Abstract

**Background/Objectives**: Accurate breast lesion segmentation using deep learning models requires precise understanding of both global contextual relevance and finer lesion structure details, which remains a challenge for existing convolutional and transformer-based approaches. This study aims to address these limitations by proposing a new segmentation model capable of improving context-aware dense segmentation tasks for ultrasound images. **Method**: We propose LTM-UNet, a novel segmentation method integrating transformer-based encoding with state-space-driven decoding in a U-Net-style framework. The architecture utilizes an efficient vision transformer encoder to extract multi-scale global representations. These features are refined through an attention-guided skip-fusion mechanism incorporating spatial-channel attention preserving finer spatial details and thereby minimizes the semantic gap between encoder and decoder features. Additionally, a direction-aware decoder based on a state-space model is introduced to efficiently capture long-range dependencies and enhance relevant feature reconstruction. **Results**: Extensive experiments on benchmark ultrasound medical imaging datasets demonstrate the effectiveness of the proposed method. The model achieves dice-score coefficients of 82.41% on the BUSI dataset and 86.62% on Dataset B (UDIAT), outperforming several existing segmentation approaches in both dice-score coefficient and Intersection-over-Union (IoU) metrics. **Conclusions**: The integration of efficient transformer-based global feature extraction, attention-enhanced feature fusion, and state-space-driven decoding enables LTM-UNet to effectively capture both structural details and contextual information, resulting in superior segmentation performance compared to existing methods.

## 1. Introduction

Breast cancer is one of the leading causes of mortality among women, imposing a considerable emotional and clinical burden. As the disease involves multiple factors, early detection and an accurate diagnosis are important if survival outcomes are to be improved. Curative success is still hampered by late-stage presentation and lack of an ideal therapeutic protocol. Breast ultrasound is widely favored among all the available imaging methods in clinical screening due to its non-invasive nature, low cost and ability to provide real-time visualization [1]. However, the task of isolating breast lesions from ultrasound images is challenging because adequate image contrast is not always guaranteed: speckle artifacts, shape variations in breast lesions and indistinct boundaries are some of the factors acting against the accurate delineation of breast lesions [2]. Such imaging problems are hindrances to the consistency of manual annotation, and have motivated a surge in interest in automated segmentation algorithms.

**Figure 1:**
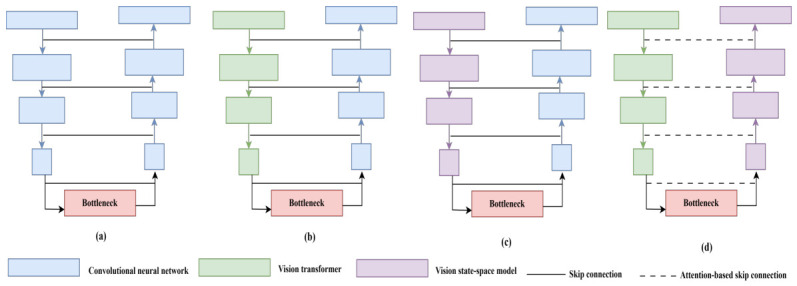
Evolution of the deep learning-based segmentation architecture. (**a**) Traditional convolutional neural network based U-Net architecture with direct skip connections; (**b**) Architecture of the transformer based U-Net architecture with direct skip connection; (**c**) Architecture of the Mamba-based U-Net architecture with direct skip connection; (**d**) Proposed transformer-Mamba architecture with attention based skip connection for breast ultrasound segmentation.

Traditional methods [3,4] mostly rely on intensity- and gradient-based descriptors to segment lesion areas, and perform reasonably well with low-resolution scans. However, handcrafted feature-extraction strategies do not consider higher-order patterns, often fail with noisy and cluttered input and also require strict assumptions regarding the visibility of lesion structure. Recently, deep learning has revolutionized the field of medical image segmentation. Figure 1 illustrates the architectural evolution of deep learning segmentation frameworks. Figure 1a shows the traditional convolutional neural network (CNN) based U-Net, employing convolution encoder–decoder pathways with skip connections, which effectively captures local features but lacks global contextual modeling. Figure 1b depicts the architecture of a vision transformer-based framework (TransUNet-style), which employs an encoder with self-attention mechanisms to model long-range global dependencies, albeit at quadratic computational cost. Figure 1c illustrates a hybrid vision state-space model (VSSM) integrated design, combining sequential state-space modeling with convolutional decoding for improved computational efficiency. In contrast, our architecture in Figure 1d advances beyond all preceding frameworks by integrating a transformer encoder, VSSM decoder, and attention-enhanced skip connections, collectively achieving a balanced global–local segmentation paradigm for breast ultrasound analysis.

The U-Net [5] family, which has a convolution encoder–decoder architecture, gave rise to a number of variants, including UNet++ [6] and DeepLabv3+ [7], which have been shown to be good at creating rich hierarchical features. Later works that added attention into the U-Net skeleton, such as Att-UNet [8,9], and incorporated multi-scale fusion modules [10,11,12], further boosted segmentation accuracy in breast ultrasound. The problem is, however, that convolution kernels only have access to a small neighborhood and they are not good at reasoning about long-range dependencies or about global structure. Hence, they are less successful if asked to draw an outline of a heterogeneous or irregularly shaped tumor. To overcome these CNN-specific drawbacks, transformer-based designs were proposed. They are able to capture global context that is not captured by pure convolutional models, due to self-attention. Such global modeling can be integrated with familiar U-shaped pipelines, such as TransUNet [13] and Swin-Unet [14], and have shown promising improvements. However, transformers have challenges—they require a greater amount of memory and computation, they require large amounts of labeled data, and patch-based tokenization can lead to a loss of fine spatial information [15]. Some hybrid CNN-transformer variants [16,17] attempt to combine local and global learning and their components are generally loosely coupled, which results in suboptimal fused representations, especially for medical images with weak and low-contrast boundaries.

Recently, a third path towards modeling long-range dependencies has been taken with State-Space Models (SSMs) [18], Mamba in particular [19], which have the advantageous property of linear computational scaling. Empirically, these models can emulate the global context in a very efficient way, with a limited consumption of resources. However, they are insufficiently sensitive to local details and boundaries [15], which are very important for ultrasound lesion segmentation. Furthermore, current pipelines based on SSMs still make use of rather immature feature-fusion techniques and are just starting to incorporate complementary learning paradigms [20,21,22,23]. Looking at the evidence, it is clear that none of the three widely used paradigms—CNNs, transformers and SSMs—have conquered breast ultrasound segmentation. CNNs work well locally and poorly globally; transformers work well globally but come at a high computational cost and sacrifice local sensitivity; and SSMs work well globally, but need more efficient means of local sensitivity and multi-scale fusion. This combination of trade-offs provides an impetus for a common approach that combines the best elements of each.

Guided by the gaps highlighted above, we introduce an architecture that brings together a linear-complexity transformer encoder and a selective directional-scanning SSM decoder inside an attention-enhanced U-Net skeleton tailored for breast ultrasound segmentation. Multi-scale, globally contextual descriptors of lesion structure are produced by an encoder that uses gated linear attention. Before these features reach the decoder, a gated-attention-driven skip-fusion module refines them, keeping informative content while muting background activations and sharpening boundary cues. The decoder itself is built on an SSM block that captures long-range spatial dependencies and reconstructs segmentation maps at reduced computational cost. By weaving together global modeling, adaptive fusion, and efficient state-space reasoning, our framework achieves a balanced depiction of both global and local features. The contributions of this study are summarized below.

1. We present LTM-UNet, an architecture that combines linear-complexity transformer encoding with state-space modeling within an attention-guided U-Net backbone, providing an effective way to model long-range dependencies for medical image segmentation.
2. We design a decoder built around directional selective state-space model, which captures long-range dependencies and addresses the well-known shortcomings of conventional convolutional or attention-based decoders.
3. We introduce an attention-guided skip-fusion block grounded in residual spatial-channel attention, which adaptively reweighs encoder features using complementary channel and spatial statistics before fusion, thereby narrowing the semantic gap and strengthening multi-scale feature integration.

The rest of the paper is laid out as follows: Section 2 provides an overview of existing related works. Section 3 presents the details of the proposed methodology. Details of datasets and experiments are described in Section 4. In Section 5, we present the ablation experiments and results, along with comparisons. Finally, we conclude this work in Section 6.

## 2. Literature Review

Over the past decade, deep learning has reshaped how medical images are segmented for automated detection and diagnosis. A broad spectrum of architectures has been studied to cope with anatomical variability, low tissue contrast, and the chronic shortage of annotated data. CNNs laid the groundwork because of their strong spatial feature extraction. More recently, vision-based foundational models [24] and their successors [25] have drawn attention for capturing wider context, partially compensating for the spatial limitations of CNNs, though their computational appetite is considerable. Vision Mamba [15], which employs structured selective state-space models, has begun to offer a more attractive trade-off between speed and accuracy, especially for long-range dependency modeling in medical imaging [26]. The following subsections review work across three key categories: CNN-based segmentation models, transformer-based approaches, and the newer Vision Mamba-based architectures.

### 2.1. CNN-Based Methods

Seminal CNNs have been proven to be an effective and widely-used tool for breast ultrasound (BUS) segmentation because of their hierarchical spatial feature learning. Early works like U-Net [5], UNet++ [6] and DeepLabv3+ [7] introduced multi-scale contextual information in the form of dense skip connections and atrous spatial pyramid pooling to the encoder–decoder template. Similarly, CE-Net [27] used dense atrous convolution and multi-kernel pooling to recover spatial information lost due to downsampling, in addition to using residual encoders to strengthen feature representation. Although dense skip connections and deep supervision have been shown to close the gap between semantic information contained in encoders and decoders and facilitate multi-scale fusion, they also introduce complexity and redundancy to such designs, which are therefore less efficient than more structured fusion approaches. This was further extended with UNet 3+ [28], where full-scale skip connections were added along with deep supervision to effectively integrate low- and high-level features across different resolutions. All these improvements contributed to improved segmentation quality, especially for imagery with objects of various scales.

In more recent CNN-based breast ultrasound segmentation (BUS), attention and more elaborate schemes of feature fusion have been leveraged. Convolutional block attention module (CBAM)-based frameworks [29] combine backbone networks like ResNet, DenseNet, and EfficientNet with attention modules to direct the network to focus on the regions of interest in a tumor, while reducing background noise. This direction of research shows that refinement via attention can be useful for enhancing both accuracy and interpretability. Similarly, DDRA-Net [30] combines dual-channel residual attention with depth-wise separable convolutions to extract subtle lesion features and maintain stability when dealing with the heterogeneity of tumors.

Another strand of the work focuses on multiple scales and the use of shallow features. To preserve the fine detail in shallow feature extraction, MLFEU-Net [31] combines a low-level enhancement block with a parallel multi-scale fusion module. Similarly, DPNet [32] integrates global context and local detail with a feature-aggregation module and both spatial and channel attention. Asym-UNet [33] enhances this approach with a multi-branch residual encoder, external attention, and specific boundary modules to highlight lesion boundaries, and DCSAU-Net [34] uses split-attention with deeper feature extraction to enhance multi-scale representations. ConvMixer and multi-scale attention gates are incorporated into a convolutional pipeline in CMU-Net [35] to expand the scope of contextual modeling, but the representational power of CMU-Net is still limited by the convolution-based global mixing in its architecture. All these models are inherently CNN-based and have no explicit modeling of global patterns, and thus are not robust if there is anatomical variation. Moreover, attention-augmented U-Nets (e.g., Att-UNet [8] and its application to ADU-Net [9]) introduce skip connections that have global context modules and progressive refinement stages to enhance the transmission of information between them. In ResU-KAN [36], the receptive field is broadened by combining residual attention with ASPP for enhancing multi-scale extraction, but it yet fails to efficiently capture long-term dependency on small datasets.

Despite these contributions, CNN-based BUS models are still inherently limited by their use of local kernels which cannot easily model long-range dependencies and global structure—these are crucial when lesions are complex and heterogeneous. While attention modules and multi-scale fusion help to alleviate this problem to some extent and at the cost of increased model complexity, they rarely address the dependency problem on a global scale.

### 2.2. Transformer-Based Methods

The ability to naturally model long-range dependencies and the global context makes transformer-based segmenters a viable alternative to conventional CNN models in BUS. The first works to demonstrate this were the TransUNet [13] and the Medical Transformer [37], both of which enabled transformers to be inserted into U-shaped architectures with CNN decoders to maintain finer spatial detail. These models clearly outperformed their convolutional predecessors when global reasoning was important. In order to broaden the generalization, SSFormer [38] proposed adding a pyramid transformer encoder, but the problem of attention dispersion and insufficient local feature modeling still remained. Under weak supervision, ScribFormer [39] adds multi-branch fusion to incorporate both global and local cues and the triple-branch design shows effectiveness at the expense of increased computational complexity.

Subsequently, efforts focused on hierarchical and multi-scale transformer architectures. An example of such an approach is the fully transformer-based encoder–decoder introduced in Swin-Unet [14], using shifted windows in order to jointly model local and global interactions. To fuse multi-scale context, DS-TransUNet [40] introduced dual-scale encoding, and to fuse local and global views, HiFormer [41] adopted a dual-branch method, with one branch consisting of CNN and the other branch consisting of a transformer. These attention mechanisms and fusion schemes have been improved in recent years for breast ultrasound segmentation. BCT-Net [42] combines dual-level attention with supervised contrastive learning, for instance, in order to focus on the discrimination of features and the accuracy of segmentation. EMGANet [43] combines edge-aware modules with multi-scale group-mix attention in order to manage the ambiguity of ultrasound image boundaries and speckle noise, and to achieve state-of-the-art results on a number of datasets. Hybrid CNN–transformer methods have also experienced significant progress; for instance, CSAU-Net [44] introduces cross-scale attention into skip connections to effectively fuse both global context and fine details at minimal cost, and SSTrans-Net [17] captures channel-wise long-range dependencies using a clever shifted-window attention mechanism to boost accuracy without significantly increasing computational requirements. Also, multi-level attention, as shown in MLFAN [10], can enhance the representation of features and facilitate the localization of lesions using hierarchical transformer encoders.

Despite these advances, transformer-style segmenters have a number of intrinsic limitations. Patch-based tokenization suffers from the loss of fine spatial detail, which is important in order to accurately delineate the boundaries in ultrasound. Secondly, transformations are typically applied to large amounts of labeled corpora and require significant computational resources, which is typically not the case for medical imaging. Third, in the fusion of CNNs with transformers, CNN–transformer hybrids are created in an attempt to balance the two model branches, but often fusion is not optimal. These concerns drive the development of global context modeling architectures, which at the same time preserve local details and are computationally efficient.

### 2.3. Vision Mamba-Based Methods

In recent years, SSM, especially the Mamba family [19], has proven to be a promising paradigm for medical image analysis. In contrast to CNNs or transformers, SSMs [18] can capture long-range dependencies in linear time, making them well suited to dense prediction tasks such as segmentation. In this regard, early methods like VM-UNet [20] integrated VSSM blocks within a U-shaped backbone to efficiently incorporate global context information and achieve competitive performance at significantly lower computational cost than transformer-based methods. A direction of follow-up work has been to make the interaction between local and global feature extraction within SSM frameworks tighter. Hybrid SS-Conv-SSM blocks were proposed in MedMamba [26] for combining convolutional local extraction with state-space modeling for long-range dependencies. Building on this, CDMamba [45] introduces a scaled residual ConvMamba block and an adaptive global–local fusion mechanism to further enhance discrimination of features in dense prediction tasks, further highlighting the importance of jointly modeling global context and local details. Later, multi-scale and feature fusion have been brought further by a variety of segmenters based on SSM. SMM-UNet [11] stacks the Selective Fusion Mamba (SF-Mamba) and Multi-Scale Fusion Mamba (MF-Mamba) modules, and tailors fusion strategies to the characteristics of lesions to achieve strong segmentation performance with minimal parameters. H-vmunet [22] introduces the idea of high-order selective scans to reduce global-level redundancy in model learning while retaining local feature learning. To achieve the goal of reducing redundancy and parameters, RM-UNet [23] introduces residual VSSM blocks and rotational SSM modules, which not only boost the ability to extract channels but also have lightweight footprints. SegMamba-V2 [46] extends the paradigm to 3D medical segmentation, using tri-orientated spatial Mamba blocks along with hierarchical scaling strategies to effectively model volumetric data.

However, there are still some weaknesses in the segmenters based on SSMs. Firstly, although they are able to adapt to long-range dependency well, they are poorer at handling fine-grained local structure, which is important for the task of boundary delineation in breast ultrasound [20]. Second, many of the current designs of SSM are pure state-space or loosely coupled hybrid designs [21], which do not optimize complementary feature spaces. Thirdly, despite being light in computation, existing fusion strategies of SSMs are still relatively immature, especially in the case of multi-scale medical segmentation. The observations indicate that for a demanding task such as breast ultrasound segmentation, using an SSM framework alone is unlikely to be sufficient, and thus there is an interest in using other paradigms along with SSMs. This is the basis of the proposed framework, which combines a transformer-based global semantic modeling encoder with an efficient long-range dependency learning decoder that is based on an SSM, and uses gated attention fusion to preserve fine-grained local detail.

## 3. Methodology

### 3.1. Overview

**Figure 2:**
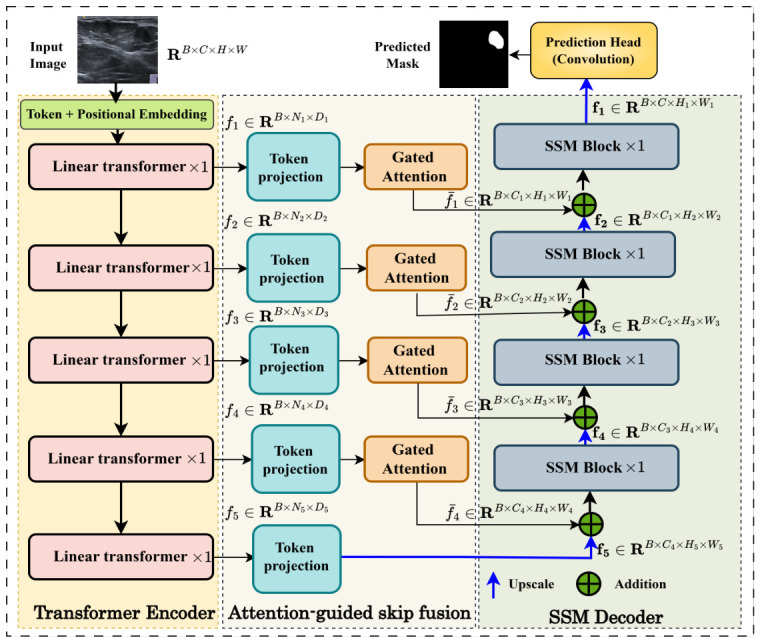
Architecture of LTM-UNet, where a linear transformer encoder generates hierarchical token features $f_{1}-f_{5}$, which are transformed into spatial maps via token-to-map projection. These features are selectively fused through attention-enhanced skip connections (gated attention) and integrated into a hierarchical SSM-based decoder with progressive upsampling and residual aggregation. The final segmentation map is produced through a convolutional prediction head.

Figure 2 shows the architecture of the proposed segmentation model. The overall design is motivated by the complementary strengths and limitations of the existing paradigms. While transformers are well-suited for learning long-range relationships, they provide little inductive bias for locality, whereas convolutional decoders excel at capturing spatial detail, but are limited in reasoning globally. We connect these two in our model and also add state-space modeling, which enables global reasoning to be achieved efficiently. In particular, this network combines a transformer encoder with a state-space-based decoder, with multi-scale representations of tokens generated using a linear vision transformer. These tokens are re-shaped into spatial feature maps and then refined step by step by attention-guided skip fusion and Mamba-based decoding blocks for joint modeling of global and local structure. The pipeline consists of several stages, given an input $X\in R^{H\times W\times C}$. First, the vision encoder provides hierarchical token representations on a number of scales. The token embeddings are subsequently reshaped into two-dimensional feature maps via token-to-map transformation. These maps are projected into different channel dimensions, producing a hierarchy of multi-scale features. Attention-based skip connections then bridge the semantic gap between encoder and decoder features by re-calibrating encoder activations using both channel- and spatial-wise attention, before fusing them with the decoder stream. The decoder reconstructs high-resolution feature maps progressively through a stack of Mamba-based blocks. Finally, the refined feature map is projected into the segmentation space and up-sampled to the original resolution to deliver the prediction. Each stage is detailed below.

### 3.2. Transformer Encoder

**Figure 3:**
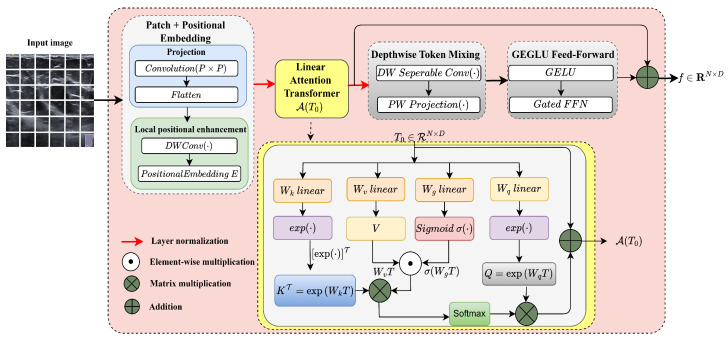
Detailed structure of the linear transformer encoder block, where input images are transformed into token embedding via convolution patch projection and local positional enhancement. The tokens are processed using a gated exponential linear attention mechanism, followed by depth wise token mixing and gated GELU (GEGLU) feed-forward layers with residual normalization, enabling efficient local–global feature modeling.

Departing from conventional transformer architectures that rely entirely on global attention, the encoder proposed here builds inductive bias and an attention mechanism tailored for dense prediction. Figure 3 outlines the linear-complexity vision transformer module that sits inside the encoder. The block augments the usual transformer tokenization with local positional enhancement, gated exponential linear attention, and stage-wise refinement, so that long-range dependencies can be modeled efficiently without erasing fine-grained local structure. Given an input image $X\in R^{H\times W\times C}$, the encoder first partitions it into non-overlapping patches of size P × P. Each patch is then projected onto a D-dimensional embedding space using a convolutional projection, as defined in Equation (1).

(1) $$T_{0}=\mathrm{Flatten}\left({\mathrm{Conv}}_{p\times p}\left(X\right)\right),\;T_{0}\in R^{N\times D}$$

where $N=\frac{H}{P}\times \frac{W}{P}$ denotes the total number of tokens. To compensate for the spatial information lost during patchification, a local positional enhancement module realized through depth-wise convolution DW(·) injects neighborhood context into every token. A learnable positional embedding $E\in R^{N\times D}$ is then added to $T_{0}$ to retain global spatial ordering.

(2) $$T_{0}=T_{0}+DW\left(T_{0}\right),T_{0}=T_{0}+E$$

Standard self-attention scales quadratically with the number of tokens. To circumvent this, we use a gated exponential linear attention mechanism that approximates the softmax with linear complexity while strengthening feature selectivity. For an input token $T\in R^{N\times D}$, the query, key, value, and gating projections are computed as

(3) $$Q=\phi \left(W_{q}T\right),K=\phi \left(W_{k}T\right),V=\left(W_{v}T\right)\;\mathrm{and}\;G=\sigma \left(W_{g}T\right)$$

where the element-wise positive map ϕ(·)=exp(·) ensures stability and σ(·) is a sigmoid gating function. The attention map is then produced via Equation (4), written as

(4) $$A\left(T\right)=\frac{Q(K^{\tau }\left(V⊙G\right))}{Q(K^{\tau }1)+\varepsilon }$$

Element-wise multiplication is denoted by the product symbol ⊙ and epsilon ε by a small constant added for numerical stability. This construction yields complexity that is linear in the sequence length and modulates features adaptively through gating. Because attention alone captures global dependencies, we restore local continuity through a depth-wise token-mixing module that reshapes the token sequence into a spatial map, applies a depth-wise separable convolution M(·), and follows it with a point-wise projection in Equation (5). The operation strengthens local feature aggregation without materially increasing computational cost.

(5) $$\hat{T}=PW\left(M\left(T\right)\right)$$

**Figure 4:**
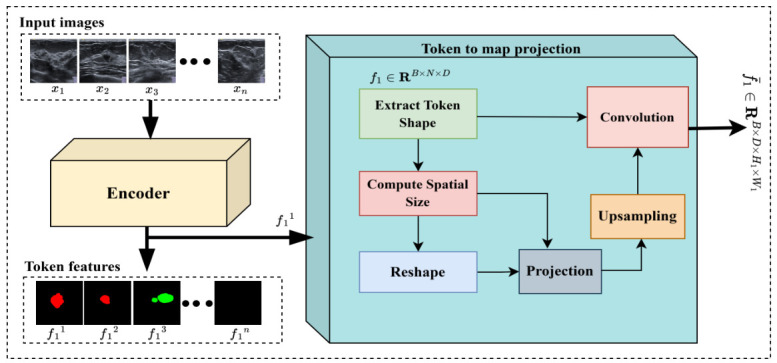
Illustration of the token-to-map projection module, where encoder-generated token features are reshaped into spatial feature maps. The token sequence is first converted to its spatial structure, then projected and upsampled, and finally refined through convolution to produce feature maps compatible with convolutional decoding.

Rather than relying on a standard MLP, we adopt a GEGLU feed-forward network, which raises representational capacity through multiplicative interactions, as expressed in Equation (6). The design allows the network to learn richer feature transformations than the linear layer alternative.

(6) $$F\left(T\right)=\left(W_{1}T\right)⊙\mathrm{GEGLU}\left(W_{2}T\right)$$

where F(·) denotes the feed-forward operator. Each transformer block combines attention and the feed-forward module within a residual scheme, as shown in Equation (7). Token sequences are passed through a stack of L such blocks. For an input sequence $T\in R^{N\times D}$, the block computes

(7) $$\hat{T}=T+A\left(LN\left(T\right)\right)\;\mathrm{and}\;T^{\prime }=\hat{T}+F\left(LN\left(\hat{T}\right)\right)$$

with A(·) representing the attention operator and LN(·) layer normalization. Standard transformers are weak in local inductive bias, which is detrimental for segmentation. Our encoder addresses this implicitly by combining convolutional patch embedding with structured token interactions so that global dependencies travel through attention while local spatial continuity is maintained throughout the convolutional embedding and hierarchical processing stages. This hybridization lets the encoder represent lesion boundaries and fine detail more faithfully than purely attention-driven counterparts. Multi-stage token features ${\left\{T_{i}\right\}}_{i=1}^{5}$ are collected from five semantic layers in order to facilitate the dense skip-guided decoding, while leaving the feature-dimension interface unchanged. The resulting representation becomes increasingly more semantically enriched: shallow layers contain low-level textures, while high-level semantic global context is encoded in deeper layers. Because the transformer output is a sequence, tokens are converted back to spatial feature maps in order to decode. A token-to-map projection module TokenMap(·) is used in Figure 4. Given token features $f_{i}\in R^{N_{i}\times D_{i}}$, where $N_{i}$ denotes the number of tokens and $D_{i}$ the embedding dimension of the $i^{\mathrm{th}}$ stage encoder, the spatial resolution is first inferred as $N_{i}=H_{i}\times W_{i}$., and the token sequence is reshaped to a 2D feature map as per Equation (8).

(8) $$F_{i}=\mathrm{TokenMap}\left(f_{i}\right)\in R^{C_{i}\times H_{i}\times H_{i}}$$

To align feature representations with the decoder requirements, a linear projection followed by upsampling is applied, and the resulting feature map is further refined using a convolutional layer. This transformation preserves the semantic richness of transformer features while restoring spatial structure, enabling effective multi-scale feature fusion in subsequent stages.

### 3.3. Attention-Guided Skip Fusion

Early encoder features mostly carry low-level spatial detail, whereas deeper decoder features carry high-level semantics. Directly fusing the two often introduces semantic inconsistency and degrades performance. To bridge this gap, we adopt an attention-guided skip-fusion mechanism centered on a hybrid Spatial-Channel Attention (SCA) module.

**Figure 5:**
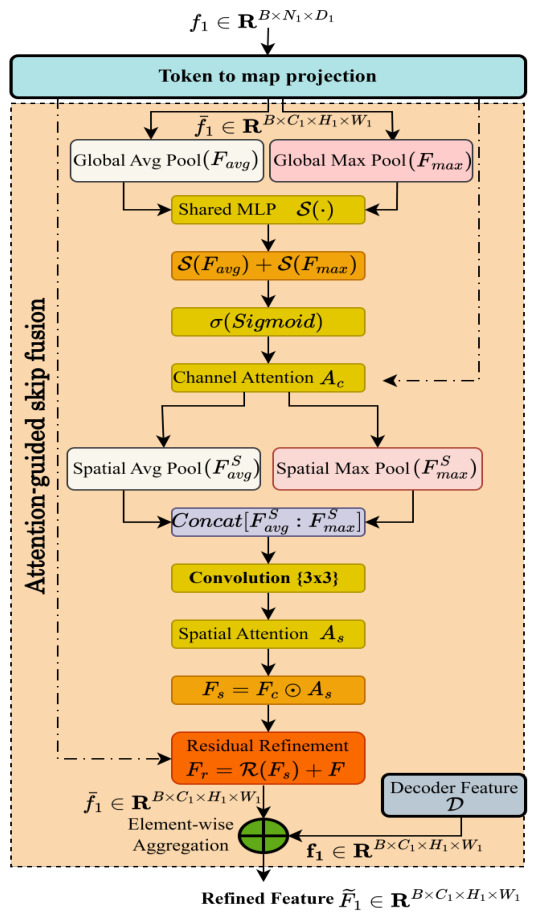
Proposed attention-guided skip-fusion module, integrating dual-statistics channel attention, spatial attention, and residual refinement to adaptively recalibrate encoder features before fusion with decoder representations, enabling improved multi-scale feature integration.

Figure 5 shows the layout of the proposed skip-fusion block, which produces refined features by jointly processing decoder-stage features alongside encoder-stage token features.

Unlike conventional skip connections that simply concatenate or add encoder and decoder features, the SCA approach refines encoder features adaptively before fusion, so that only context-relevant information is propagated. The SCA module performs re-calibration in both the channel and spatial domains, selectively emphasizing discriminative features and suppressing irrelevant ones. Given an encoder feature map F, channel attention is computed from complementary global descriptors obtained via global average pooling GAP(·) and global max pooling GMP(·), as expressed in Equation (9). Both descriptors are passed through a shared transformation S(·), realized as a lightweight multi-layer perceptron, defined in Equation (10).

The channel-refined feature map $F_{c}$ is obtained by using Equation (11). In this dual-statistics scheme, the model can consider both the global contextual trend (from average pooling) and the salient activations (from max pooling), which can provide more powerful channel-wise weighting.

To improve spatial discriminability further, SCA is based on spatial attention, which is obtained by combining the information from channels. $F_{c}$ (in Equation (12)) is subjected to spatial average pooling and spatial max pooling. The maps are then concatenated and passed to a convolution (Equation (13)).

Finally, the spatially refined features are retrieved using Equation (14). This mechanism focuses the network’s attention on key spatial areas such as lesion boundaries and salient structures, which contribute to segmentation accuracy.

(9) $$F_{avg}=\mathrm{GAP}\left(F\right),\;F_{max}=\mathrm{GMP}\left(F\right)$$

(10) $$A_{c}=\sigma \left(S\left(F_{avg}\right)+S\left(F_{max}\right)\right)$$

(11) $$F_{c}=F⊙A_{c}$$

(12) $$F_{avg}^{s}=\frac{1}{C}\sum_{i=1}^{C}F_{c}^{\left(i\right)},\;F_{max}^{s}=\max_{i\in [1,C]}F_{c}^{\left(i\right)}$$

To ensure that the attention mechanism does not destroy the important information, a residual refinement block R(·), denoted as a simple lightweight convolutional transformation, is added, which is defined in Equation (15). The residual connection is used to maintain the original feature distribution while allowing the network to learn fine-grained representation. The encoder feature $F_{r}$ is then fused with the obtained decoder feature D in an element-wise manner, as shown in Equation (16).

(13) $$A_{s}=\sigma \left(\mathrm{Conv}\left(\left[F_{avg}^{s}:F_{max}^{s}\right]\right)\right)$$

(14) $$F_{s}=F_{c}⊙A_{s}$$

(15) $$F_{r}=R\left(F_{s}\right)+F$$

(16) $$\tilde{F}=F_{r}+D$$

This formulation provides the decoder with features that are both semantically aligned and enhanced by context, and therefore result in better reconstruction quality and scale-invariant features. To enhance the multi-scale feature integration, the SCA-based skip-fusion mechanism adaptively refines the features of the encoder in both channel and spatial domains to introduce more discriminative and context-aware representations which are crucial for robust segmentation in the decoder.

### 3.4. SSM Decoder

**Figure 6:**
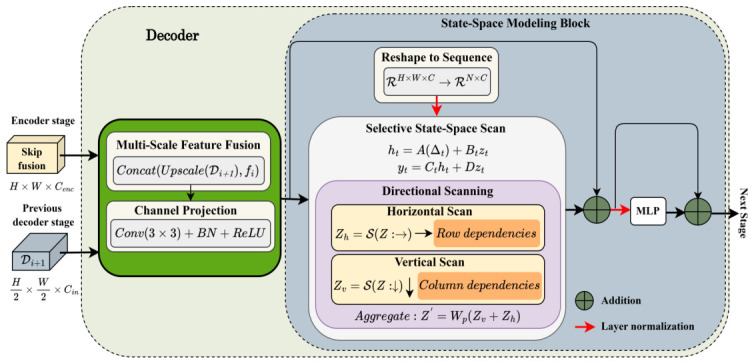
Architecture of the decoder with feature fusion and state-space modeling block, where spatial features are reshaped into sequences and processed via selective state-space scanning with directional (horizontal and vertical) dependencies, followed by MLP and normalization for enhanced contextual feature learning. (Black arrow represents information flow between connecting blocks.)

In segmentation, the decoder plays a pivotal role in rebuilding high-resolution predictions from compressed representations. Classical convolutional decoders capture local detail well but cannot reason globally, whereas attention-based decoders model global context at a steep computational price. To overcome both shortcomings, we adopt a state-space-driven decoding paradigm in which global dependencies are modeled efficiently through selective state-space dynamics. Figure 6 shows the decoder building block, which receives inputs from the matching encoder stage via skip fusion and from the previous decoder stage. Each decoder stage is followed by an up-sampling step, except for the final stage where the features are mapped to the native spatial resolution. The decoder couples multi-scale feature fusion with directional, selective state-space modeling.

At each decoding stage, the module receives the up-sampled decoder feature $D_{i+1}$ and the corresponding refined encoder feature $F_{i}$ produced by attention-gated skip fusion. To ensure spatial alignment, the up-sampled decoder feature ${\tilde{D}}_{i+1}$ is obtained through a scaling operation. These features are then concatenated with the encoder feature $F_{i}$ to form an intermediate representation $X_{i}$. A convolutional projection reduces the channel dimensionality and produces a compact representation ${\tilde{X}}_{i}$ that serves as the decoder input, as defined in Equation (17).

(17) $${\tilde{X}}_{i}=\delta \left(BN\left(Conv\left(X_{i}\right)\right)\right)\;\mathrm{where},\;X_{i}=Concat\left({\tilde{D}}_{i+1},F_{i}\right)$$

The fused features are subsequently processed by a Vision Mamba block, which models long-range dependencies through selective state-space formulation. The spatial feature map ${\tilde{X}}_{i}\in R^{C_{i}\times H_{i}\times H_{i}}$ is first reshaped into a sequence $Z_{i}\in R^{N\times C}$ with N=H×C. The state-space dynamics are defined by Equation (18).

(18) $$h_{t}=A\left(\Delta_{t}\right)h_{t-1}+B_{t}x_{t},\;y_{t}=C_{t}h_{t}+Dx_{t}$$

Here $h_{t}$ denotes the hidden states, A(·) is a learnable transition matrix, $\Delta_{t}$ is an input-dependent discretization step, $B_{t}$ and $C_{t}$ are input-dependent projection matrices, and *D* is a skip-connection parameter. For stability and efficient learning, the transition matrix is parameterized as A=−exp(·), which guarantees stable dynamics. The discretization step is bounded, as shown in Equation (19), supporting adaptive temporal scaling.

(19) $$\Delta_{t}=\sigma \left(W_{\Delta }x_{t}\right)$$

To extend state-space modeling to 2D data, a directional scanning strategy processes feature sequences in both horizontal and vertical directions. The resultant outputs are then aggregated and passed through a learnable projection matrix $W_{p}$, represented in Equation (20):

(20) $$Z^{\prime }=W_{P}\left(Z_{h}+Z_{v}\right)$$

where $Z_{h}$ is the horizontal scan and $Z_{v}$ is the vertical scan. This formulation lets the model encode row-wise dependencies through horizontal scanning and column-wise dependencies through vertical scanning, capturing spatial structure across the feature map. To further enrich expressiveness, the Vision Mamba block adds feed-forward transformations and residual connections, as defined in Equation (21). The refined sequence is then reshaped back into the spatial form $D_{i}\in R^{1\times H\times W}$ using the native convolution operation as a prediction head producing single channel output.

(21) $$Z^{\prime \prime }=Z+\mathrm{MLP}\left(\mathrm{LN}\left(Z\right)\right),\;Z^{\prime \prime \prime }=Z^{\prime \prime }+\mathrm{MLP}\left(\mathrm{LN}\left(Z^{\prime \prime }\right)\right)$$

This decoder design delivers efficient long-range modeling through the state-space formulation. Combining convolutional fusion with structured spatial reasoning preserves local detail while maintaining global consistency, both of which are necessary for dense prediction tasks.

### 3.5. Loss Function

We use a composite objective function $L_{com}$ for training LTM-UNet, which ensures regional consistency, pixel-wise classification accuracy and boundary preservation. The function consists of three complementary losses: dice loss, weighted binary cross-entropy loss and a stable boundary-aware loss. To directly optimize the segmentation overlap we resort to using the dice loss that compares the predicted and ground truth regions. The dice loss $L_{dice}$ is defined as in Equation (22), where ε is a small constant to stabilize the numerical computation for a prediction $\hat{Y}$ and the ground truth *Y*. Since it is not per-pixel based, dice loss works well in situations of class imbalance.

(22) $$L_{dice}=1-\frac{2\sum_{i}{\hat{Y}}_{i}Y_{i}+\varepsilon }{\sum_{i}Y_{i}+\sum_{i}{\hat{Y}}_{i}+\varepsilon }$$

To boost the pixel-wise classification and to penalize misclassified regions, binary cross-entropy loss $L_{BCE}$ with logits is used (Equation (23)). In our implementation, we used a higher weightage for foreground pixels so as to achieve higher sensitivity for the minority class.

(23) $$L_{BCE}=-\left[\omega \cdot Y\log\left(\sigma \left(P\right)\right)+\left(1-Y\right)\log\left(1-\sigma \left(P\right)\right)\right]$$

P is the raw prediction logits, σ(·) is the sigmoid function, and the positive class weight is ω to combat imbalance. $L_{dice}$ provides global overlap supervision and $L_{BCE}$ provides fine-grained pixel supervision and stabilizes training. We introduce a boundary-aware loss $L_{boundary}$ that is based on edge consistency to obtain better segmentation along object boundaries. The Sobel operator is used to obtain vertical $E_{x}$ and horizontal edge maps $E_{y}$, which are then normalized as $\hat{E}$. The boundary loss is then calculated by using Equation (24). This formulation prevents problems with square-root operations, and focuses on edges to produce sharper and more accurate edges.

(24) $$L_{boundary}={∥{\tilde{E}}_{pred}-{\tilde{E}}_{gt}∥}_{1}$$

Lastly, the composite loss $L_{com}$ is obtained by combining $L_{dice}$, $L_{BCE}$ and $L_{boundary}$, as explained in Equation (25). Taking into account the complementary strengths of each term, the model jointly optimizes the accuracy of region, pixel, and boundary, thus providing high-quality and robust segmentation.

(25) $$L_{com}=\lambda_{1}L_{dice}+\lambda_{2}L_{BCE}+\lambda_{3}L_{boundary}$$

These losses can be viewed as a whole, the composite loss is robust to the class imbalances seen in medical segmentation and covers overlap, pixel-level supervision and edge alignment.

## 4. Datasets and Experimental Details

This section presents an overview of the two datasets of breast ultrasound images used to validate the segmentation model. Next, the experimental details and training protocol are described and then the five performance measures are discussed.

### 4.1. Datasets Description

Two breast ultrasonic imaging datasets are used in this study to assess the performance of the proposed model. Table 1 shows the distribution of sample sizes for the dataset used in this paper. The BUSI dataset [47] is a publicly available breast ultrasound segmentation benchmark consisting of 780 ultrasound images collected from 600 female patients. The dataset contains three categories: 437 benign images, 210 malignant images, and 133 normal images. Pixel-level lesion segmentation masks are provided for benign and malignant cases, while normal images do not contain lesion annotations. In this work, normal cases were treated as background-only masks during segmentation training and evaluation in order to improve false-positive suppression and background discrimination capability.

Dataset B (UDIAT) [48] is another publicly available breast ultrasound lesion segmentation dataset containing 163 images collected from different patients, including 110 benign and 53 malignant lesion cases. The dataset contains expert-annotated lesion masks and includes substantial variability in lesion size, shape, boundary visibility, and image contrast. The average image resolution is approximately 760 × 570 pixels. Both datasets were resized and normalized prior to training to ensure consistent input representation for the proposed LTM-UNet framework.

### 4.2. Implementation Details

This subsection describes the implementation, training protocol, and evaluation strategy used to validate the proposed framework. All experiments were carried out in PyTorch 2.7 with CUDA 11.8. Training and evaluation were performed on a workstation equipped with an Intel Xeon W-225 CPU and an NVIDIA RTX A4000 GPU. To keep experimental conditions consistent across datasets, every ultrasound image was resized to a fixed spatial resolution of 256 × 256. For experimental evaluation, each dataset was split into training and testing sets in an 88:12 ratio. The training set was further split into 80:20 for final training and validation sets. This partitioning yielded 546 training, 137 validation and 97 testing images in the case of the BUSI dataset, and 114 training, 29 validation and 20 testing images in the case of Dataset B (UDIAT). Because dataset B (UDIAT) is relatively small, extensive augmentation and cross-validation were employed to minimize overfitting and improve generalization capability. To ensure robust and reproducible evaluation, all experiments were conducted using a 5-fold cross-validation strategy, with shuffled random image-wise partitioning and a fixed random seed of 42. Since patient-level identifiers are not consistently available for all publicly accessible samples, the dataset partitioning was performed at the image level rather than at the patient level. The model is trained for 120 and 200 epochs on BUSI and dataset B, respectively, using end-to-end optimization with the composite loss function described in Section 3.5. The loss coefficients were fixed at $\lambda_{1}=0.5$ for the DSC term and $\lambda_{2}=0.4$ for the BCE term, while the boundary weight $\lambda_{3}$ was progressively raised across epochs within [0,0.2]. This curriculum-style weighting allows the model to learn coarse region segmentation first and then progressively refine boundary precision, ultimately improving segmentation quality.

### 4.3. Evaluation Metrics

To quantitatively assess the proposed model, five primary metrics are used: dice score coefficient (DSC), Intersection over Union (IoU), precision (PR), recall (RC), and specificity (SP). DS measures the overlap between predicted and ground-truth regions, while IoU reports the ratio of intersection to union, giving complementary views of segmentation accuracy and consistency. Precision, recall, and specificity round out the picture. Precision quantifies the fraction of predicted foreground pixels that are correct, reflecting the model’s ability to avoid false positives and contain over-segmentation. Recall captures how well the network detects all true foreground pixels, emphasizing the importance of minimizing false negatives. Specificity tracks the fraction of background pixels correctly identified and so indicates how effectively the model suppresses spurious activations and maintains clean boundaries, an especially important property for highly imbalanced datasets where background dominates. Together these metrics provide a balanced view of both region-level correctness and structural precision.

## 5. Experimental Results

This section reports a sensitivity analysis of the main components of LTM-UNet, together with the influence of composite loss on performance. We then compare our model against existing state-of-the-art segmentation methods and finally assess robustness.

### 5.1. Ablation Experiments

#### 5.1.1. Architectural Impact

**Figure 7:**
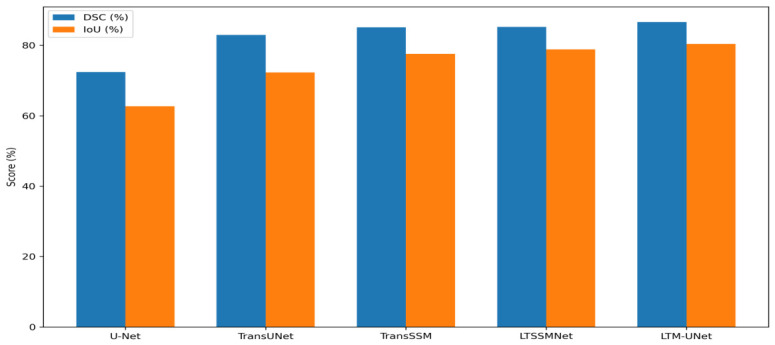
Architectural ablation of LTM-UNet showing the contribution of the linear transformer encoder, SSM-based decoder, and attention-guided skip fusion in terms of DSC and IoU.

To assess the contribution of each component, we conducted a structured architectural study of the BUSI dataset, progressing from a baseline convolutional encoder–decoder and gradually incorporating transformer-based encoding, SSM decoding, and attention-guided skip fusion. Performance was tracked using DSC and IoU. Figure 7 reports the outcomes for the architectural variants on dataset B (UDIAT).

The baseline mirrors a standard U-Net, a CNN encoder–decoder with simple skip connections. It reaches a DSC of 72.73% and an IoU of 62.70%. Replacing the CNN encoder with a vision transformer while keeping the convolutional decoder and simple skips produces a TransUNet-style configuration that pushes the metrics to 82.96% DSC and 72.33% IoU. Modeling long-range dependencies across the entire image evidently sharpens semantic understanding and lesion localization, although the CNN decoder still struggles to propagate global context during reconstruction. Swapping the CNN decoder for an SSM-based decoder, while retaining the simple skips, produces TransSSMNet, which reaches 85.10% DSC and 77.62% IoU, a meaningful gain attributable to better modeling of long-range spatial dependencies during reconstruction, and leading to cleaner lesion delineation and fewer fragmented predictions. Replacing the transformer with a linear transformer while keeping the SSM decoder (LTSSMNet) yields 85.25% DSC and 78.86% IoU. Finally, adding the attention-guided skip-fusion module on top of LTSSMNet achieves the best result of 86.62% DSC and 80.42% IoU. The attention-driven skip fusion plays a central role in selectively emphasizing informative encoder features, suppressing noisy or redundant activations, and preserving fine boundary detail. Together with the improved transformer encoder, it produces a markedly better alignment between encoder and decoder representations. Taken together, the progressive improvements confirm that LTM-UNet successfully fuses transformer-based global representation, state-space spatial modeling, and attention-guided feature integration into a coherent design that delivers superior segmentation performance.

#### 5.1.2. Impact of Composite Loss Function

**Figure 8:**
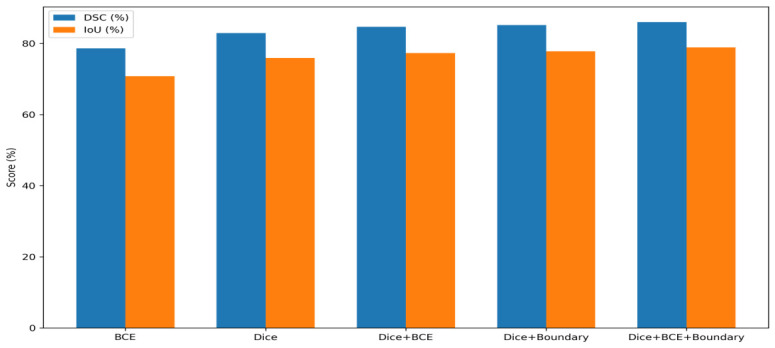
Comparison of different loss configurations. The hybrid loss combining Dice, BCE and boundary-aware supervision achieves the best overlap performance.

A series of ablations were performed on BUSI using five loss settings, accounting for variations in optimization objectives in order to explore the effect of these on segmentation performance measured by DSC and IoU. The results are summarized in Figure 8.

Training with only $L_{dice}$ that targets overlap directly yields 77.61% DSC and 70.83% IoU. Dice loss is helpful in the case of class imbalance, but used alone it can lead to unstable gradients and pixel-wise calibration issues, especially in the early stages of training. With only $L_{bce}$ which is pixel-wise classification, it achieves 75.9% DSC and 69.92% IoU. BCE provides good optimization and well-calibrated probabilities, yet is not as robust to imbalance and fragments the predictions in the case of small lesions, therefore dragging down the overlap-based metrics. When the two are hybridized as $L_{dice}+L_{bce}$, the performance is increased to 80.7% DSC and 77.3% IoU. The hybrid loss combines the global region alignment with the pixel-level accuracy and is superior to both of them. When adding the boundary term $L_{boundary}$ in the hybrid, the performance is the best, with 82.41% DSC and 80.45% IoU. The boundary term explicitly penalizes the error at object boundaries, thus favoring sharp and accurate boundaries, which is important in medical imaging; for instance, where accurate delineation of lesions is relevant for diagnosis. The supervisory edge signal enhances the localization of the edges and their structural consistency, as well as the quality in fine detail. In general, the experiments validate the need for a composite loss to optimize overlap, local pixel accuracy and boundary precision.

#### 5.1.3. Impact of Attention-Guided Skip Fusion

To evaluate the effectiveness of attention-based skip fusion, we ran comparative experiments with different skip-fusion strategies. The results are summarized in Table 2. The baseline simply concatenates encoder and decoder features without any attention refinement. Although such conventional skips preserve spatial information, they often introduce semantic inconsistencies between low-level encoder activations and high-level decoder features, producing noisy propagation and false-positive activations. The proposed skip-fusion mechanism addresses this by combining channel and spatial attention recalibration.

The numbers indicate that combining spatial and channel attention, rather than fusing encoder and decoder features directly, brings substantial improvements compared to the baseline. The improvement stems from better semantic alignment and from suppressing irrelevant background activations before they are passed to the decoder, leading to more faithful mask reconstruction. The mechanism filters out noise and smooths gradient flow during training. In particular, the multi-scale refinement branches improve lesion boundary continuity and reduce the number of disconnected false-positive segments.

#### 5.1.4. Impact of Data Augmentation

Breast ultrasound datasets, especially dataset B (UDIAT), are typically small and prone to overfitting. To boost generalization, we incorporate an ultrasound-oriented augmentation strategy into training. The ablation contrasts no augmentation, plain geometric augmentation, intensity-only augmentation, and the proposed ultrasound-aware augmentation. Table 3 lists the outcomes. Within the training pipeline, all images and masks are resized to 256 × 256, during validation and testing only resizing and normalization are applied. During training, we use a lightweight, ultrasound-friendly augmentation set consisting of horizontal flipping, mild spatial transformation, and intensity perturbation. Specifically, the pipeline applies a horizontal flip with probability (p=0.5), a shift of up to 3%, a scale variation of 5%, a rotation of up to $10^{\circ }$, brightness/contrast adjustment up to 10%, and ImageNet-style normalization. These transformations are deliberately moderate to avoid introducing anatomically unrealistic distortion.

The results indicate that training without augmentation produces the weakest validation performance, a clear signal of overfitting on the small dataset. Geometric augmentation strengthens robustness against minor variations in probe placement, lesion orientation and anatomical alignment with horizontal flipping and mild shift/scale/rotation in particular encouraging spatially invariant lesion representations, while preserving anatomical plausibility. Intensity augmentation then improves performance further by mimicking acquisition-time variation in brightness, contrast, and tissue echogenicity, all of which differ across machines, operators, and settings. The ultrasound-oriented strategy delivers the strongest overall performance by combining spatial and appearance diversity. Moderate transformation limits keep the lesion morphology anatomically valid while broadening the diversity of training samples, so the model becomes less dependent on specific lesion positions, contrast distributions, or local texture patterns. Additionally, we conducted an experimental analysis to investigate the impact of varying the number of encoder-decoder stages on the proposed model’s performance. The quantitative results corresponding to different network depths are presented in Table S1 of the Supplementary Materials. Overall, the ablation confirms that this augmentation pipeline raises DSC and IoU by curbing overfitting, improving generalization, and increasing tolerance to realistic variability in ultrasound imaging.

### 5.2. Model Training Results

**Figure 9:**
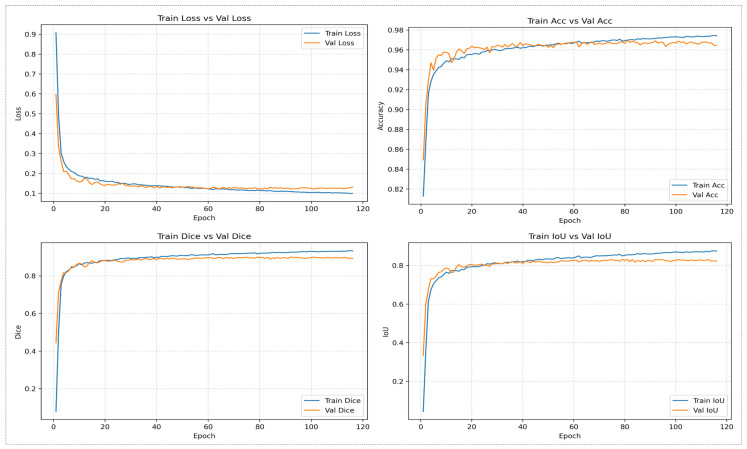
Training and validation performance curves on the BUSI dataset, illustrating the convergence behavior of loss, accuracy, DSC, and IoU across epochs, demonstrating stable learning and strong generalization of the proposed model.

The training dynamics on BUSI, as presented in Figure 9, show that the convergence of training is stable and generalization is good. The training and validation losses decrease quickly in the initial epochs, indicating the quick learning of discriminating features, and then stabilize, with only minimal fluctuation after about 40–50 epochs.

The two curves are in close proximity to each other, indicating that there is no significant overfitting. The accuracy shows a similar trend, with a gradual increase (≈96–97%) for both types of data, suggesting reliable pixel-level classification. The metrics based on overlaps present a consistent picture: DSC is increasing rapidly and stabilizing at 0.90–0.92 for training and 0.88–0.90 for validation, whereas IoU is converging towards ∼0.85–0.88 for training and ∼0.82–0.84 for validation.

The small but consistent gap between training and validation curves across DSC and IoU suggests mild generalization error but no severe overfitting. Overall, the convergence behavior across all four metrics confirms that the model effectively captures both global context and fine-grained boundary details, achieving robust and reliable segmentation performance on the BUSI dataset.

**Figure 10:**
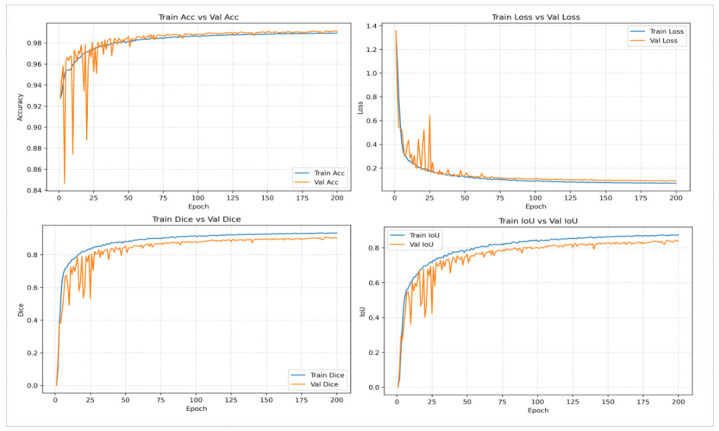
Training and validation performance curves on Dataset B (UDIAT), illustrating the convergence behavior of loss, accuracy, DSC, and IoU across epochs, demonstrating stable learning and strong generalization of the proposed model.

The training behavior on Dataset B (UDIAT) in Figure 10 demonstrates effective learning with slight instability during the early training phase, followed by strong convergence and generalization. The loss curves show a rapid decline in both training and validation loss within the initial epochs, indicating efficient feature learning. However, noticeable fluctuations in the validation loss during the first ∼30 epochs suggest sensitivity to data variability or class imbalance. As training progresses, both curves stabilize and closely align, confirming that the model converges well without significant overfitting. A similar trend is observed in the accuracy curves, where both training and validation accuracy increase steadily and saturate at high values (∼98–99%). The early oscillations in validation accuracy further reflect initial instability, but the eventual overlap with training accuracy indicates improved generalization. The DSC and IoU curves reinforce this observation: both metrics show sharp improvements in the early epochs, despite validation fluctuations, and gradually stabilize at strong values. The small gap between training and validation performance across these metrics suggests minimal generalization error. Overall, despite early-stage volatility, the model achieves stable convergence on dataset B (UDIAT) and effectively captures both region-level and boundary-level features. The final performance indicates robust segmentation capability, with strong overlapping of scores and consistent agreement between training and validation trends.

### 5.3. OverfittingPrevention and Generalization

The breast ultrasound dataset used in this work is limited in size, particularly for dataset B (UDIAT), and therefore susceptible to overfitting. This challenges the model’s generalizability, so we adopt several complementary strategies to address it. We perform all experiments using five-fold cross-validation, and the reported results correspond to the average performance across all iterations. This protocol reduces dependency on a specific train–test partition and provides a more reliable estimate of generalization capability. Secondly, an aggressive ultrasound-oriented data augmentation strategy is adopted during training, including horizontal flipping, geometric transformations, and brightness/contrast perturbations. These augmentations increase sample diversity while preserving anatomical realism and lesion morphology.

Furthermore, the linear transformer encoder incorporates local positional enhancement and depth-wise token mixing, introducing local inductive biases that reduce the data requirements typically associated with transformer architectures. This design improves learning efficiency on limited medical datasets. Finally, a composite Dice–BCE–Boundary loss is utilized to jointly optimize region overlap, pixel-wise classification accuracy, and boundary consistency. This multi-objective supervision acts as an additional regularization mechanism by preventing over-specialization to a single optimization criterion. The training and validation curves were monitored throughout training. As shown in Figure 9 and Figure 10, the validation loss closely follows the training loss, while the DSC and IoU curves show only a small, stable gap between training and validation performance. This behavior indicates stable convergence and suggests that the proposed model generalizes well without significant overfitting.

### 5.4. Comparison with State-of-the-Art Methods

Table 4 presents the comparative results of benchmark methods on BUSI and Dataset B (UDIAT).

As shown in Table 4, LTM-UNet achieves mean DSC values of 81.38 ± 1.03 and 85.37 ± 1.25 on the BUSI and dataset B (UDIAT) datasets, respectively, over five-fold cross validation, outperforming competing methods such as Asym-UNet [33] and CSAU-Net [44]. The model also achieves the highest PR (89.63%) and a good RC (88.56%), suggesting a good balance in segmentation with few false positives and false negatives. While SP for MLFEU-Net [31] is slightly higher (99.48%), our model’s specificity is also very competitive (99.45%) and in the overlap metrics, which are more in line with segmentation quality, our model clearly performs better. Finally, LTM-UNet performs better than the transformer-based baselines TransUNet [13] and SSFormer [38] when compared in a visual manner, which shows the usefulness of combining the efficiency of transformers with state-space modeling and attention-guided fusion. Our model is also the best performing among models like MLFEU-Net [31] and Asym-UNet [33], with the highest IoU (80.42%) and DSC (86.62%) on dataset B (UDIAT). LTM-UNet achieves 92.56% PR and 87.42% RC, which, while slightly lower than the CSAU-Net [44] and Asym-UNet [33], is still in good balance, ensuring reliable detection of lesions. Its SP (98.36%) is competitive too, indicating a good level of background discrimination. Unlike TransUNet [13], our model augments global context modeling with state-space decoding and attention-guided fusion and richer local feature representation, which further boosts model performance compared with TransUNet, which already benefits from global context modeling. The statistical evaluation of the proposed LTM-UNet using five-fold cross-validation across benchmark datasets is reported in Table 4. While these findings reflect consistent and reliable segmentation performance, a formal statistical comparison with certain baseline methods remains difficult, as their corresponding publications do not provide measures of variability such as standard deviations or confidence intervals. Overall, the results validate the effectiveness of LTM-UNet for segmentation tasks, demonstrating its ability to achieve higher accuracy and a more balanced precision–recall curve, which directly stems from the hybrid design and combines transformer-based global modeling, state-space efficiency, and attention-guided skip fusion.

**Figure 11:**
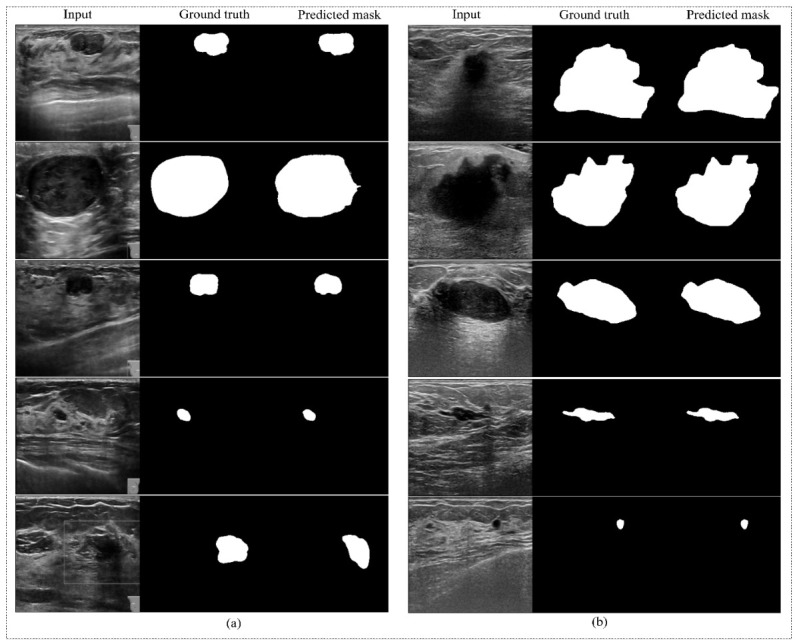
Segmentation outputs of LTM-UNet. (**a**) Visualization of the segmented mask on the BUSI dataset. (**b**) Visualization of the segmentated mask on dataset B (UDIAT).

As shown in the Figure 11, our model is effective in segmenting breast lesions of varying size, shape and in varying structural condition. The segmented masks for test set BUSI and dataset B (UDIAT) are given in Figure 11a and Figure 11b, respectively. The predicted masks are highly consistent with ground-truth annotations, both on small and large lesions; the model is able to capture the fine-grained structure of the lesion and maintain smooth boundaries and anatomically plausible masks. Normal images in the BUSI dataset do not contain lesion annotations or foreground segmentation masks; therefore, during segmentation training, the corresponding masks for normal cases were treated as all-background (zero-valued) masks. This enables the model to learn both lesion–region localization and suppression of false-positive activations in healthy breast ultrasound images. During evaluation, predictions generated for normal images were compared against these background masks, thereby contributing to specificity and false-positive assessment. Hence, normal cases improve the robustness of the segmentation model by encouraging discrimination between lesion and non-lesion regions under realistic clinical conditions. This is particularly important in breast ultrasound imaging, where speckle noise and heterogeneous tissue appearance can otherwise lead to false lesion predictions. The model provides compact, spatially coherent lesion masks, and does not suffer from the problems of scattered or disconnected predictions that many conventional approaches do. Especially in irregular and non-uniform lesions, the boundary delineation capability is evident and the contours of predicted and actual lesions are in good agreement. All these observations imply that the lightweight transformer encoder, the attention-guided skip-fusion module, and the SSM-based decoder are effective in strengthening global context understanding and local structural reconstruction.

### 5.5. Model Complexity

Comparative analysis of the efficiency–performance trade-offs of the different segmentation architectures is presented in Table 5 in terms of the number of parameters, computational cost (FLOPs), and inference times. However, the traditional designs, including Att-UNet [8] and UNet++ [49] fall in the moderate-to-high end of computation requirements. Attention-UNet [8] has the highest FLOPs (72.81 G) and latency (37.17 ms), despite having fewer parameters than transformer models, and UNet++, while light on parameters (9.16 M), still requires significant computation, indicating inefficient feature aggregation. The two models based on transformers—TransUNet [13] and SSFormer [38]—show different characteristics: TransUNet [13] has the highest number of parameters (105.32 M) and is memory-hungry, whereas SSFormer [38] significantly lowers FLOPs (13.56 G) while maintaining the parameter overhead to a reasonable level. More complex hybrids like HiFormer [41], ScribFormer [39] and MFAN [10] try to achieve a balance between complexity and speed: Hi-Former has the lowest FLOPs (12.51 G) and inference time (19.57 ms), while MFAN and ScribFormer take intermediate approaches. However, none of these models can be considered to be optimal on all axes. LTM-UNet on the other hand, offers a good compromise between efficiency and computational cost. It has very low FLOPs (15.96 G) and is significantly lighter than most of the transformer variants, with 23.72 M parameters. It also provides the fastest inference time of 13.77 ms, indicating efficient feature processing, which is crucial for real-time applications. This profile showcases the impact of the combination of transformer-based encoding, state-space modeling and attention-guided skip fusion, all of which help to lower the computational burden and run the system faster. The complete hyperparameter configuration employed during model training is summarized in Table S2 of the Supplementary Materials. A computational complexity and parameter comparison of pure Mamba/SSM-based methods is presented in Table S3 of the Supplementary Materials. Overall, LTM-UNet strikes an appealing balance, rendering it ideal for real-time or resource-limited medical image segmentation tasks.

### 5.6. Model Generalization

Table 6 reports the cross-domain results. The proposed model achieves strong in-domain performance with IoU/DSC of 80.45/82.41 on BUSI and 80.42/86.62 on dataset B (UDIAT), confirming effective learning of dataset-specific characteristics. When trained on BUSI and tested on Dataset B (UDIAT), the model retains a comparable DSC (82.13%) but shows a noticeable drop in RC (73.87%), suggesting reduced sensitivity to unseen domain variation. Conversely, training on dataset B and testing on BUSI produces a larger decline (IoU 67.04%, DSC 72.30%), highlighting a moderate domain-shift effect in that direction. PR and SP remain comparatively high across all settings, indicating that localization stays consistent and false positives stay limited. Qualitative cross-domain segmentation results of the proposed LTM-UNet, evaluated across the BUSI and Dataset B (UDIAT) datasets, are presented in Figure S1 of the Supplementary Materials. Taken as a whole, the model demonstrates reasonable generalization, although the residual performance gap in cross-domain scenarios reflects inherent differences in dataset distribution. Although LTM-UNet, when trained on dataset B and tested on BUSI, highlights the challenges of generalizing across different datasets, variations in imaging conditions and dataset characteristics likely contribute to this performance gap. Further validation on larger and more diverse multi-center datasets is needed to better assess the model’s generalizability.

### 5.7. Limitation

Although LTM-UNet demonstrates better performance on the BUSI and dataset B (UDIAT), the experimental evaluation is limited to globally hosted breast ultrasound datasets acquired under specific imaging conditions. Since ultrasound images exhibit significant variations across clinical locations, device variations, operators and patient population, the generalization capability of the model must be further validated on large multi-center and multi-vendor datasets. The next phase of this will focus on cross-institutional evaluation and domain adaptation strategy to further improve generalization and clinically applicable models across various imaging environments. Another limitation of the present work relates to how the dataset was divided for training and testing. Partitioning was performed at the image level, which does not guarantee that all images of a given patient are confined to a single subset. As a result, patient-specific information may inadvertently overlap across splits, leading to optimistic performance estimates. Subsequent studies will incorporate patient-wise data division along with independent validation cohorts to establish a more reliable measure of the model’s real-world applicability.

## 6. Conclusions

This work introduces a segmentation framework that integrates efficient transformer-based global context modeling with state-space learning and attention-enhanced skip connections, together within a U-Net paradigm. By tackling the limited global awareness of conventional CNNs, the high computational cost of vision-based foundational models, and the modest local sensitivity of SSMs, the proposed architecture achieves a balanced and efficient representation of both local and long-range dependencies. Empirical results on BUSI and dataset B (UDIAT) show that LTM-UNet outperforms many state-of-the-art methods across IoU, DSC, PR, and RC. The model also produces sharp boundary delineations and maintains a favorable precision–recall trade-off, attesting to its value for breast ultrasound image segmentation. The framework offers a solid foundation for hybrid segmentation models, and its modular nature opens several directions for advancing intelligent, efficient medical image analysis.

## Figures and Tables

**Table 1:** Distribution of sample sizes for BUSI and Dataset B (UDIAT).

Datasets	Benign	Malignant	Normal	Total
BUSI	437	210	133	780
Dataset B	110	53	-	163

**Table 2:** Effect of the proposed attention-guided skip fusion module on breast ultrasound segmentation performance.

Configuration	Channel Attention	Spatial Attention	DSC (%)	IoU (%)	PR (%)	RC (%)
Baseline (Direct skip)	×	×	78.23	78.52	83.95	85.53
Channel Attention	✓	×	81.20	79.62	85.25	86.26
Spatial Attention	×	✓	80.66	78.38	86.53	86.72
Both	✓	✓	82.41	80.45	89.63	88.56

**Table 3:** Ablation study on the effect of different augmentation schemes on Dataset B (UDIAT).

Experimental Settings	Resize	Horizontal Flip	Shift/Scale/Rotate	Brightness/Contrast	DSC (%)	IoU (%)
W/O augmentation	✓	×	×	×	82.63	76.58
Geometric augmentation	✓	✓	✓	×	83.52	79.24
Intensity augmentation	✓	×	×	✓	82.41	80.40
Ultrasound-oriented augmentation	✓	✓	✓	✓	86.62	80.42

**Table 4:** Segmentation results comparison between LTM-UNet and existing works. Results are reported on a five-fold cross-validation on BUSI and Dataset B (UDIAT) datasets. The arrow indicates that a higher value is desirable. Best restlts are highlighted in bold.

Datasets	Methods	Year	IoU (%) ↑	DSC (%) ↑	PR (%) ↑	RC (%) ↑	SP (%) ↑
	U-Net [5]	2015	63.06	76.35	74.29	78.78	96.73
	UNet++ [6]	2018	63.55	80.54	82.75	74.21	97.58
	TransUNet [13]	2021	60.57	79.30	83.68	71.61	95.01
	SSFormer [38]	2022	64.75	79.27	-	77.06	96.53
	CMU-Net [35]	2022	65.74	78.29	-	75.70	95.71
BUSI	DCSAU-Net [34]	2023	66.56	75.14	-	80.99	-
	SMM-UNet [11]	2024	68.80	81.12	-	-	-
	MFAN [10]	2025	78.14	79.73	-	88.13	98.07
	Asym-UNet [33]	2025	71.85	80.46	87.09	78.99	96.45
	MLFEU-Net [31]	2025	71.86	80.82	85.12	82.80	**99.48**
	CSAU-Net [44]	2025	70.92	81.22	84.49	83.57	96.01
	Res-UKAN [36]	2025	67.74	79.92	-	-	-
	LTM-UNet (ours)	–	**80.45**	**82.41**	**89.63**	**88.56**	99.45
	U-Net [5]	2015	62.70	72.43	76.38	78.17	98.84
	UNet++ [6]	2018	67.05	75.07	74.05	83.48	98.85
	TransUNet [13]	2021	72.33	82.96	81.26	85.33	96.23
Dataset B (UDIAT)	MLFEU-Net [10]	2025	77.49	86.44	87.89	**87.48**	**99.43**
	CSAU-Net [44]	2025	72.91	83.72	90.71	85.80	98.60
	Asym-UNet [33]	2025	76.42	84.04	**93.56**	80.63	98.23
	LTM-UNet (ours)	–	**80.42**	**86.62**	92.56	87.42	98.36

Results indicated in the table were obtained from the standard publication. Direct statistical comparisons with some existing methods are limited by the absence of variance measures in their published results.

**Table 5:** Comparative analysis of segmentation models in terms of parameter count, computational complexity (FLOPs), and inference time, demonstrating the efficiency of the proposed LTM-UNet. The results for LTM-UNet are reported on NVIDIA RTX A4000 with an image dimension of 256 × 256. Best results are highlighted in bold.

Method	Parameters (M)	FLOPs (G)	Time (ms)	DSC (%)
Attention U-Net [8]	34.88	72.81	37.17	60.17
UNet++ [49]	**9.16**	34.65	36.17	60.38
TranUNet [13]	105.32	38.52	32.45	79.79
SSFormer [38]	66.22	**13.56**	29.49	79.27
Hiformer [41]	25.21	12.51	19.57	82.38
ScribFormer [39]	50.43	35.91	23.01	75.06
MFAN [10]	29.44	31.88	19.29	79.73
Asym-UNet [33]	94.64	52.25	–	80.46
LTM-UNet	23.72	15.96	**13.77**	**82.41**

Results indicated in the table were obtained from the standard publication.

**Table 6:** Cross-domain evaluation of the proposed LTM-U-Net model trained and tested across BUSI and Dataset B (UDIAT) datasets. The results demonstrate the model’s generalization capability under domain-shift conditions. The arrow indicates higher values are desirable.

Train Dataset	Test Dataset	IoU (%) ↑	DSC (%) ↑	PR (%) ↑	RC (%) ↑	SP (%) ↑
BUSI	BUSI	80.45	82.41	89.63	88.56	99.45
BUSI	Dataset B	77.87	82.13	88.08	73.87	96.63
Dataset B	Dataset B	80.42	86.62	92.56	87.42	98.36
Dataset B	BUSI	67.04	72.30	83.06	66.52	91.25

## Data Availability

The BUSI dataset used in this paper is obtained from publicly available datasets while Dataset B (UDIAT) can be made available upon valid request. The BUSI dataset is available at https://scholar.cu.edu.eg/?q=afahmy/pages/dataset. The Dataset B (UDIAT) can be accessed at https://helward.mmu.ac.uk/STAFF/m.yap/dataset.php. The relevant source codes are available at https://github.com/shivpratap10/LTM-UNet, all accessed on 8 June 2026.

## Supplementary Materials

The following supporting information can be downloaded at https://www.mdpi.com/article/10.3390/diagnostics16121888/s1, Figure S1: Qualitative cross-domain segmentation results of the LTM-UNet under two settings: BUSI (Train) → Dataset B (Test) (left) and Dataset B (Train) → BUSI (Test) (right); Table S1: Experimental results of varying depth of encoder-decoder on model’s performance; Table S2: Hyperparameter configuration of the proposed LTM-UNet model integrating transformer-based encoding and state-space decoding with attention-guided skip fusion; Table S3: Comparative analysis of pure Mamba/SSM based segmentation models in terms of image size, encoder-decoder stages, parameter count, computational complexity (FLOPs), and datasets, demonstrating the efficiency of the proposed LTM-UNet. The results of [20,21] are compared with LTM-UNet in Table S3 of supplementary material.
