# Supplementary material for: LTM-UNet: Linear Transformer–Mamba with Attention-Based U-Net for Context-Aware Breast Ultrasound Image Segmentation
Source: Diagnostics (Basel). 2026 Jun 17;16(12):1888. doi: 10.3390/diagnostics16121888 (PMC13298203; doi:10.3390/diagnostics16121888)
Supplement: Supplementary file 1 [file diagnostics-16-01888-s001.zip › diagnostics-4280963-supplementary.pdf]

Supplementary Martials

LTM-UNet: Linear Transformer-Mamba with Attention-based U-Net for Context-Aware Breast Ultrasound Image Segmentation

Impact of number of stages

Table S1. Experimental results of varying depth of encoder-decoder on model's performance.

Depth	Architecture	Decoder	Acc. (%)	IOU (%)	Dice score (%)
**2**	ViT + VSSM	2x2	85.6	71.2	72.3
**3**	ViT + VSSM	3x3	96.4	75.6	78.1
**4**	ViT + VSSM	4x4	99.4	80.4	82.4
**5**	ViT + VSSM	5x5	98.5	79.5	81.8

We study the effect of architectural depth on the model's performance. For this study, we assess our model's performance by varying the depth values (d) and changing the number of encoder and decoder stages. We measure the performance of models starting from depth 2, which consists of two encoding and decoding stages, and up to 5, which comprises five encoding and decoding stages. Table 3 displays the performance of the models at various depths. We achieved the best performance at depth of 4. Expanding the depth beyond four does not yield further improvements and slightly degrades performance, likely due to overfitting and excessive feature compression.

Specifications of hyperparameters and descriptions.

Table S2. Hyperparameter configuration of the proposed LTM-UNet model integrating transformer-based encoding and state-space decoding with attention-guided skip fusion.

Category	Parameter	Value	Description
**Input**	Image size	256 × 256	Resized input resolution
	Channels	3	RGB ultrasound images
**Encoder (Transformer)**	Patch size	16	Tokenization granularity
	Embedding dimension	320	Feature dimension
	Number of stages	4	Multi-scale feature extraction

	Dropout	0.2	Regularization
	Drop path	0.2	Stochastic depth
**State-Space Decoder (TSSM)**	d_state	16	State dimension
	d_conv	4	Local convolution width
	Expansion ratio	2	Channel expansion
	MLP ratio	2.0	Feed-forward scaling
	Decoder stages	4	Hierarchical upsampling
**Skip Fusion (Attention Guided)**	Fusion type	Attention-based	Adaptive feature merging
	Reduction ratio	4	Channel compression
**Feature Processing**	Channel reduction	{64, 96, 160, 256}	Multi-scale mapping
	Normalization	LayerNorm + BatchNorm	Stabilization
**Output Head**	Output channels	1	Binary segmentation
	Logit scaling	6.0	Stabilized logits
	Activation	Sigmoid (in loss)	Probability mapping
**Loss Function**	Dice weight	0.5	Region overlaps
	BCE weight	0.4	Pixel-wise classification
	Boundary weight	0.05–0.15	Edge refinement
**Optimization**	Optimizer	AdamW	Adaptive optimization
	Learning rate	5×10^{-5}	Initial LR
	Weight decay	1×10^{-4}	Regularization
	Gradient clipping	0.3	Stability
**Scheduler**	Type	Cosine Annealing	LR decay
	Minimum LR	1×10^{-6}	Lower bound
**Training Setup**	Batch size	8	Per iteration
	Epochs	120 (Dataset B)	Total training

		200 (BUSI)	
	K-fold	5	Cross-validation
	Random seed	42	Used for Python, NumPy, PyTorch, and CUDA
**Data Augmentation**	Flip	Horizontal & Vertical	Spatial diversity
	Rotation	±10°	Robustness
	Normalization	ImageNet stats	Standardization
**Inference**	Threshold	0.3	Binary mask cutoff

Visualizations of cross-dataset segmentation masks.

Figure S1 shows qualitative cross-domain segmentation performance under two settings: BUSI → Dataset B (left) and Dataset B → BUSI (right). Each row shows the input image, ground truth mask, and predicted mask. In the BUSI → Dataset B case, the model successfully captures lesion regions with shapes and locations closely matching the ground truth. Although minor boundary smoothing and slight size discrepancies are visible, the predictions remain consistent, indicating good generalization from BUSI to Dataset B.

In contrast, the Dataset B → BUSI scenario exhibits noticeable degradation. The predicted masks are smaller and occasionally miss portions of the lesion, reflecting reduced sensitivity. This aligns with the quantitative drop in recall observed earlier. However, the predictions still maintain reasonable localization with limited false positives. Overall, the figure highlights that the model generalizes better from BUSI to Dataset B than vice versa, emphasizing the impact of domain shift and dataset characteristics on segmentation performance.

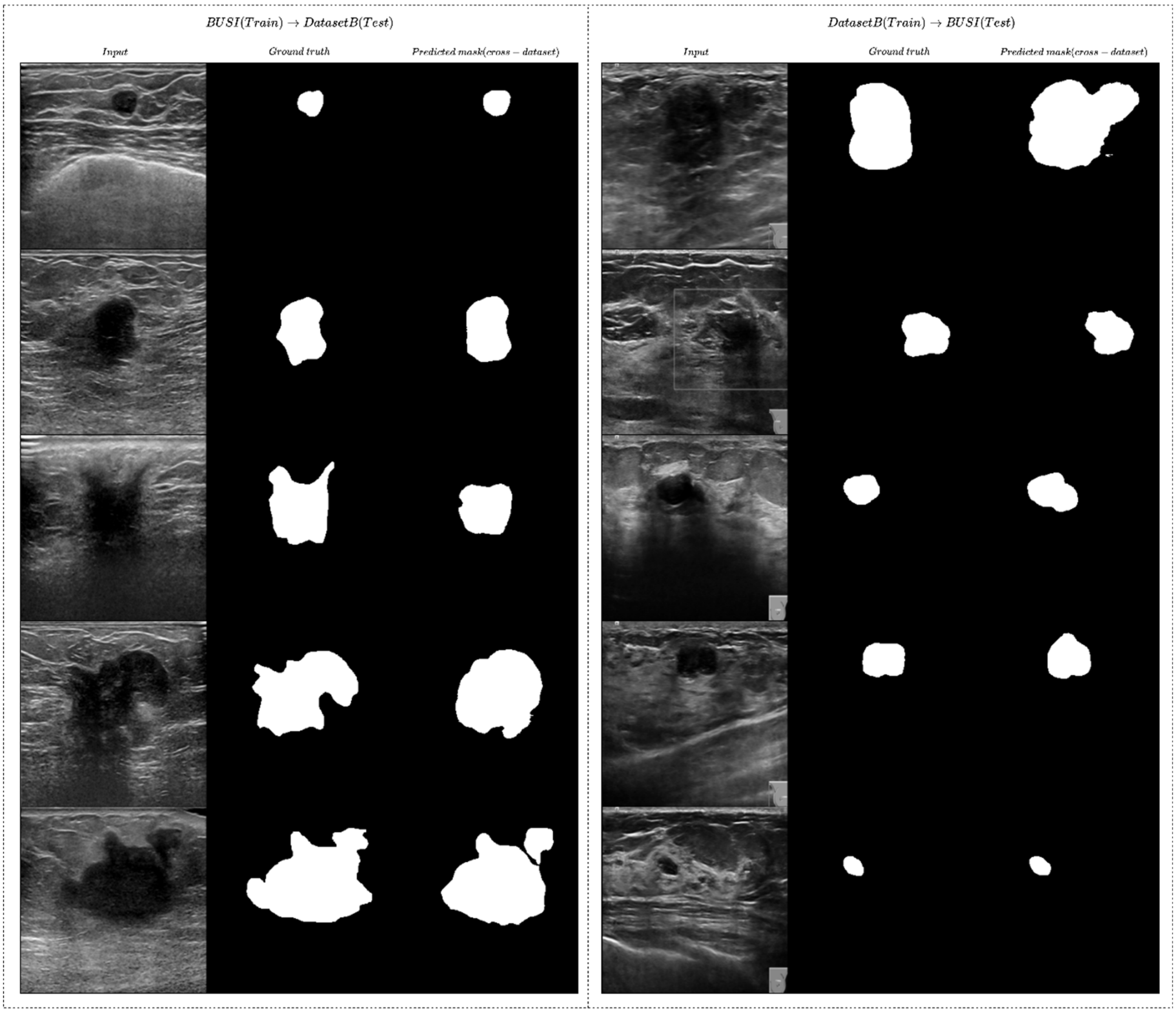

Figure S1. Qualitative cross-domain segmentation results of the TSSM-UNet under two settings: BUSI (Train) → Dataset B (Test) (left) and Dataset B (Train) → BUSI (Test) (right). Each row shows the input ultrasound image, corresponding ground truth mask, and predicted segmentation. The model demonstrates strong generalization in the BUSI → Dataset B setting with accurate lesion localization and shape consistency, while performance degradation is observed in the reverse direction, indicating the impact of domain shift.

Computational complexity and parameters comparison of the pure Mamba/SSM based methods.

Table S3 compares the architectural complexity of recent Mamba-based segmentation frameworks with the proposed LTM-UNet. VM-UNet and Swin-UMamba employ deeper symmetric encoder–decoder configurations ({2,2,2,2-2,2,2,2}), resulting in higher parameter counts of 27.53M and 28M, respectively. In contrast, LTM-UNet adopts a lighter decoder design ({2,2,2,2-1,1,1,1}), reducing the parameter count to 23.72M while maintaining efficient long-range dependency modeling. Although VM-UNet reports lower FLOPs, it was evaluated on dermoscopic datasets with different modality characteristics. Swin-UMamba exhibits the highest computational complexity (18.9G FLOPs) despite using a smaller input size of 192×192. LTM-UNet achieves a balanced trade-off between architectural efficiency and segmentation capability on challenging breast ultrasound datasets (BUSI and Dataset B), demonstrating that lightweight decoder-stage optimization can effectively reduce model complexity without sacrificing representational strength.

Table S3. Comparative analysis of pure Mamba/SSM based segmentation models in terms of image size, encoder-decoder stages, parameter count, computational complexity (FLOPs), and datasets, demonstrating the efficiency of the proposed LTM-UNet.

Model	Image Size	Encoder-Decoder stages	Parameters	Flops	Dataset
VM-UNet [1]	256x256	{2,2,2,2-2,2,2,2}	27.53M	4.35G	ISIC 2017 and 2018
Swin-UMamba [2]	192x192	{2,2,2,2-2,2,2,2}	28M	18.9G	AbdomenMRI, Endoscopy, Microscopy Images
LTM-UNet	256x256	{1,1,1,1-1,1,1,1}	23.72	15.96	BUSI and Dataset B

1. Jiacheng Ruan, Jincheng Li, and Suncheng Xiang. 2025. VM-UNet: Vision Mamba UNet for Medical Image Segmentation. ACM Trans. Multimedia Comput. Commun. Appl. (September 2025), https://doi.org/10.1145/3767748
2. J. Liu et al., "Swin-UMamba†: Adapting Mamba-Based Vision Foundation Models for Medical Image Segmentation," in IEEE Transactions on Medical Imaging, vol. 44, no. 10, pp. 3898-3908, Oct. 2025, doi: 10.1109/TMI.2024.3508698

Data availability

The data used in this paper were obtained from publicly available datasets.

Code availability

The relevant source codes are available at

https://github.com/ shivpratap10/LTM-UNet.git
